# The Proteid Iron Preparations of National Formulary, or the N. F. Propaganda, with Some Queries and Conclusions

**Published:** 1908-08

**Authors:** 


### The Proteid Iron Preparations of National Formulary, or the N. F. Propaganda, With Some Queries and Conclusions.

[Editorial in *Critic and Guide*, June, 1908.]

INTRODUCTION.

THIS is a very important editorial. It is the most important editorial that I have written in several months. It deals with fundamental principles. It is going to present certain questions. And unless these questions are answered satisfactorily, our leaders in pharmacy, aided and abetted by some temporarily misguided physicians, will stand convicted before the world as engaged in a nasty business, injurious alike to pharmacy and medicine and criminally wrong as far as our patients are concerned.

I am entirely willing to be judged by this editorial as to my knowledge of the situation and as to my ability to reason fairly, logically and unanswerably. I am either right or wrong. If I am shown to be wrong, then I am unworthy to be a leader in medicine or in medico-pharmaceutical journalism, and the professors of pharmacy and therapeutics and the pharmacists in general who are paid subscribers to the *Critic and Guide* should show their displeasure by discontinuing their subscriptions. If, however, I am right, absolutely right, then this editorial should and will mark an epoch in American pharmacy and medicine (or rather medicinal therapeutics).

I have an unshakable belief in the invincible power of truth. It may be temporarily overshadowed by falsehood, you may succeed in filling the people's eyes with dust for a time, but after all the immortal saying of the immortal Lincoln about fooling the people remains an eternal verity.

When we have the temerity to state that some of the pharmacopeial and most of the National Formulary preparations intended as *substitutes* for well-known standard remedies are not "just as good" as the originals, that in fact some of these imitations are nasty, ill-tasting and ill-smelling concoctions (and that it is therefore wicked to mislead the physician and the pharmacist—the former to prescribe and the latter to dispense these substitutes), we are accused by some narrow-minded druggists, and some misguided or ignorant doctors, of bias. To assure our accusers that we are as free from bias as any living human can be and, that our only misfortune is, that we have a penchant for telling the truth, regardless of consequences, would be a waste of time. Let us therefore see what pharmacists themselves—and real pharmacists with laboratory facilities—have to say about some of the National Formulary preparations.

Prof. W. H. Harrison, of the Northwestern University School of Pharmacy, Chicago, read a paper before the Chicago Branch of the American Pharmaceutical Association entitled "Notes on Proteid Iron Solutions." The paper appears in *The American Journal of Pharmacy* for April, and we advise every honest physician and pharmacist to read it there in its entirety. An abstract of it also appears in the A. Ph. A. Bulletin for May. Dr. Harrison considers the three proteid iron preparations of the National Formulary: Liquor Ferri Peptonati, Liquor Ferri Peptonati cum Mangano, and Liquor Ferri Albuminati. Of the first Dr. Harrison has the following to say:

LIQUOR FERRI PEPTONATI.

"The present National Formulary formula yields a product which is a thick red-brown liquid, with a very *disagreeable gluey*[1] odor. It is clear in neither reflected nor transmitted light, and of such a colloidal nature as to render filtration impossible even under greatly increased pressure. The taste is at first pleasant, followed by a strongly *alkaline* and *ferruginous* aftertaste, which persists."

He then proceeds to show the reasons why a good preparation is impossible. The chief trouble lies in the peptone, of which it is impossible to obtain in the open market satisfactory or uniform specimens. Whether obtained from meat or fish albumin they "are prone to *rapid putrefaction* and yield iron combinations of most *offensive* odors."

Of Liquor Ferri Peptonati cum Mangano, which is openly and frankly intended as a substitute for Pepto-Mangan Gude, and on which substitute an immense amount of time and labor has been expended, the author has the following to say:

LIQUOR FERRI PEPTONATI CUM MANGANO.

"When made according to the present formula, with the materials obtainable on the market, the National Formulary preparation may be described thus:

"A dark brown *sluggish* liquid, with a most *offensive* odor, not unlike a mixture of *ammonia* and *putrefied beef extract.* Taste *alkaline, saline* and *nauseating.* It deposits after a time a *dirty white sediment,* which soon covers the bottom of the vessel.

"The finished product contains about .15 per cent. iron, .145 per cent. or less manganese, and .234 per cent. ammonium hydroxide, the latter serving the *sole purpose* of developing more *offensive* odors.

"I have prepared four samples, in each case using different samples of peptonised iron, the finished products being almost identical.

---

1. Italics ours throughout.—Ed. C. and G.

"The trouble with this preparation lies principally with the peptonised iron, and ammonium hydroxide, although there is room for improvement elsewhere.

"Of six samples of peptonised iron examined, the products of the principal manufacturers of pharmaceutical chemicals, *all* showed that *putrefaction* was in progress[1]. Of seven examined for iron content, only one showed over 5 per cent. Fe O (3.5 per cent. Fe), and this one sample has not yet been on the market under the name of peptonised iron or iron peptonate.

"At the time this work was started, but two samples of iron peptonate and none of the soluble manganese citrate were obtainable on the Chicago market.

"After some time I succeeded in collecting some direct from the manufacturers, seven samples of peptonised iron and two of soluble manganese citrate.

"These two samples of soluble manganese citrate, although bearing the same title, are *entirely different substances.*

"(1) A light red-brown powder with a strong odor of acetamide and ammonia. It is a manganese-ammonium citrate containing about 18 per cent. manganese. Incompletely soluble in water, but solution is rendered clear by standing for some time with a slight excess of ammonia.

"(2) Pearl-colored scales (evidently made after the formula of F. B. Power, Proceedings A. Ph. A., 1902, 937). Contains 13.5 per cent. manganese. It is a manganese sodium citrate freely water-soluble.

"In view of the above facts, it seems that a satisfactory preparation according to the present N. F. formula is *impossible,* although with a good sample of peptonised iron it could yield a *passable* one."

Now, gentlemen of the medical and pharmaceutical professions, please read the above carefully, very carefully. Here we have a preparation of great, thoroughly established, therapeutic value. That it is of great, thoroughly established, therapeutic value is seen from the fact that it is prescribed by physicians universally throughout the country. That it is prescribed universally is seen from the fact that every manufacturer, big or little, and every would-be pharmaceutical chemist is racking his brains and spending his time and labor in his endeavor to prepare a successful substitute for Dr. Gude's pepto-mangan. And what

---

1. Dr. Harrison is not alone in his opinion. All pharmacists who investigated the matter think the same. Mr. M. I. Wilbert, one of our foremost pharmacists, and a member of the Council of Pharmacy and Chemistry of the American Medical Association, says: "This formula (*for Liquor Ferri Peptonati cum mangano*) directs that commercial ferric peptonate be used. This substance *at best* is *variable,* is *unstable,* and, as *usually* met with, is *decomposed* and *unfit* for use. Commercial manganese peptonate, suggested in the alternative formula, is even more unsatisfactory than the ferric peptonate." (*American Jour. of Pharmacy,* May, 1907, p. 211.)

is the result? What have we got? After hundreds and hundreds of attempts, after many years of labor, the leaders of pharmacy give us in the *third* edition of their book as a substitute for a well-known ferruginous tonic a formula, which yields in the hands of the *best* pharmacists a preparation of "a most *offensive* odor, *not unlike a mixture of ammonia and putrefied beef extract.* Taste *alkaline, saline,* and *nauseating,* and depositing after a time a *dirty white sediment!*"

Is this the aim of real professional pharmacy?

And I appeal to you all to answer this question: If you had a boy or girl, wife or mother who was very anemic and was in need of a mild, assimilable, non-irritating ferruginous tonic, would you give the original pleasant-to-eye, smell and taste—and stable pepto-mangan, or would you give the National Formulary Liquor Ferri Peptonati cum Mangano, which is physically, pharmaceutically and therapeutically *rotten* (there is no other term possible), which according to the testimony of pharmacists themselves has a *most* offensive odor, alkaline, saline and nauseating taste and becomes very quickly decomposed? Would you run the risk of ruining their stomach and making them still sicker, because the imitation product may perhaps cost ten cents cheaper? And if you would not, if in your own family you would use the genuine product, why should you treat the outside public so badly?

Dr. Harrison claims that after numerous trials he has succeeded in preparing a satisfactory solution of iron peptonate with manganese. He gives an exceedingly elaborate formula and process. Assuming this to be the case, does any one believe that one druggist in a thousand would go to all these pains to select materials of the highest quality? And does any one believe that one druggist in a thousand would succeed in making a satisfactory preparation by following Dr. Harrison's elaborate directions which it took him months to perfect? And what is it all for? And this leads us to the important question:

### WHAT IS IT ALL FOR?

Who inoculated us with this crazy substitution-mania? What obsession has taken possession of us, that no sooner has a preparation became popular no sooner as a real demand been created for it, than pharmaceutical professors and sub-professors, their assistants and sub-assistants, our manufacturers and their employes anxious for a raise, and, what is worse, our National Formula makers, begin to spend time, labor and material, in order to prepare a more or less satisfactory (?) *substitute!* As a result of this we get a hundred different imitations, all varying in color, odor, taste, chemical composition and therapeutic action, and many of them positively rank, irritating and injurious. And this is called the elevation of pharmacy and therapeutics! It is not thus in Europe. We do not hear of the English, German, French or Italian professors and pharmacopeia makers spending their time and labor in the attempted manufacture of imitations of well-known products. They spend their time and labor in *original research and investigation!*

The imitations, we said, all differ widely in character and not one of them is as good as the original. The reasons are easy to understand. The manufacturer of one or only a few specialties devotes his entire time, energy and capital to those specialties. He makes numerous experiments; he uses materials of the highest obtainable quality; he invents or instals special machinery, if necessary. All these things are entirely out of the question with the retail druggist, and even with the big general pharmaceutical manufacturer; for making several hundred to several thousand different preparations, it is impossible for him—*it does not pay him*—to devote too much time, labor and expense to an imitation of somebody else's specialty—especially as he has no reputation to gain or lose on it. Yes, the reasons are perfectly plain, why the imitations are never as good as the really worthy original additions to our therapeutic armamentarium. But while I knew a priori that this was so, I wanted to convince myself by concrete examples, by incontrovertible facts. I secured samples of practically *every* preparation which our noble pharmaceutical leaders have introduced into our Pharmacopeia and National Formulary as substitutes for well-known proprietary products. I secured samples of the "official imitations" of arsenauro, antiphlogistine, aristol, lysol, pepto-mangan, Gray's gylcerine tonic, Gardner's hydriodic acid, Fairchild's essence of pepsin, Carlsbad salts, glyco-thymoline, listerine, even of such a simple thing as resinol, and *not in one instance* was the imitation equal to the original in purity, taste, homogeneousness, stability, etc. Some of the preparations were absolutely rank, disgusting, and I could but feel contempt, mixed with indignation, against certain high moguls of pharmacy, who mislead the poor retail druggist and the unsophisticated physician into the belief, that their careless, imperfect, theoretical, extemporaneous formulae will yield products "just as good" as the standard products, which are the result, perhaps, of many years of chemical or pharmaceutical research and which are prepared in specially adapted laboratories with the utmost care.

We will now pursue another line of thought. Let us assume for a moment that after the expenditure of a lot of time and labor somebody has succeeded in preparing an imitation of some well established proprietary, which is *absolutely* "just as good"—

absolutely the same—pharmaceutically, chemically and therapeutically. Let us assume it. What has been accomplished? What has been added to pharmacy and chemistry? Nothing! Not an iota. Merely a product that has already been in existence and in use, has been duplicated by somebody else. But here somebody will be sure to interject: Why, the product has been cheapened. A product that can be manufactured by everybody is generally cheaper than a monopoly product. But to whom is the product cheaper? To the public? Any such assertion would be emphatically untrue. Just prescribe 12 ozs. or a pint of the imitations, let us say, of Liq. ferri peptonati cum mangano or Elix. gentian. glycerinat. and see how much a druggist will charge. As a matter of fact I have been told and know personally of instances where my good friends, the druggists, make it a rule to charge *more* for the N. F. preparations than for the original products. Incredible? Just try it yourself. Do you want additional testimony from an unimpeachable source? Take The American Journal of Pharmacy for May 1907, and open it to page 236. On that page you will read the following:

"Professor Remington, in the course of his remarks, strongly deprecated the reported tendency of pharmacists to *charge more* for U. S. and N. F. preparations than for corresponding proprietary preparations, and expressed the belief that practices of this kind would surely do much to discredit the propaganda and do an infinite amount of harm."

It is thus *seen*—and seen in a manner which cannot be gainsaid—that the public is not at all benefited by this U. S. P.—N. F. propaganda. Who then is benefited? The druggist? Yes, that I admit. The druggist is to a certain extent benefited by this propaganda. And nobody begrudges it to him. Eking out as he does a very poor living after working longer hours than any other tradesman or professional man, nobody, I am sure, will grudge the druggist a few extra cents profit (*provided* the imitation products are really in every respect as good as the original ones). But this being so, that is the manufacture of imitation products *not* tending to the elevation of pharmacy and chemistry and *not* being of the slightest benefit to the public, let us say so! Let us have a clear understanding as to what all this propaganda is about. Let us stop talking about the elevation of professional pharmacy, let us stop throwing dust into the eyes of the unsophisticated physician, and let us acknowledge openly and honestly that the entire N. F. propaganda is a movement instituted for the purpose of affording the druggist a larger profit on physicians' prescriptions and—if it must be said—of making substitution respectable, of giving it, so to say, an official status. Is this putting it too strong? But it is the truth, and the language of truth, said the Romans, is simple; simple, plain and direct.

A WARNING.

And here I wish to utter a word of friendly warning to the pharmacists of this country, which warning I trust will be heeded by the readers of the *Critic and Guide*. Suppose the N. F. propaganda is successful and the doctors begin to prescribe N. F. preparations instead of standard long-established products. Then the druggist must be *sure*—and this is my warning—that the preparations he dispenses are really of high merit physically (taste, odor, etc.), pharmaceutically and therapeutically. Otherwise, he will only hurt himself, the thing will act as a boomerang; the doctor's confidence in the retail druggist's ability will be shaken still further, and he will be still further strengthened in his belief that the safest thing is to prescribe brand preparations of known composition—or he will be driven into self-dispensing. Here are two actual experiences—two out of many that I could relate. A physician was in the habit of prescribing large quantities of Hayden's Viburnum Comp. The druggist to whom most of the prescriptions used to go thought it wise to do some missionary work with the doctor, showing him circulars about nostrums, etc., and urged him to prescribe the N. F. substitute for H. V. C., which he claimed was superior. The doctor finally, half persuaded, wrote a prescription for Viburnum compound N. F. The druggist prepared it extemporaneously and dispensed it. The woman complained to the doctor that the medicine did not taste like the other times, made her sick at the stomach and didn't do her any good. The doctor, as he told me, then sent the N. F. to ——, continued to prescribe as formerly and the missionary druggist is now getting fewer prescriptions from him than formerly. The second case is one in which a druggist dispensed a muddy ill-smelling, strongly alkaline mixture instead of peptomangan which the doctor had prescribed, and as a result lost almost his entire prescription trade; for the doctor was one of those who looked at the substitution business very seriously and took pains to tell the members of his medical society that the druggist O. was a substitutor.

Yes, make sure, when you do create a demand for U. S. P. and N. F. preparations, that you are able to supply the demand. For it is a well-known fact that *not* 5 per cent. of the druggists in the country are capable of preparing even the half-way complex preparations of the U. S. P. and N. F. (such as the organic iron preparations, effervescent salts, etc.).

We are not alone in our opinion that the N. F. propaganda is not the best thing in the world. Some prominent *pharmacists*

think the same way. Take *The American Journal of Pharmacy* (June, 1907). On page 296 you will find a report of a paper entitled "Practical Results with N. F. Preparations" read before the Philadelphia College of Pharmacy. In discussing that paper, a prominent pharmacist, Mr. D. J. Thomas, "was inclined to question the advisibility of pursuing this line of work at the present time, thinking that *the rank and file of pharmacists were not prepared to meet the demand* for U. S. P. and N. F. preparations. He recounted some experiences that had come to his attention that appeared to indicate that pharmacists in his locality, like pharmacists in other sections, had been remiss in their duty to themselves and their customers, and had not kept themselves posted on the progress of pharmacy along the more practical lines. He also called attention to several formulas that *when followed exactly did not give satisfactory preparations.* Among these he enumerated the glycerinated elixir of gentian and the cataplasm of kaolin."

This editorial could be drawn out so as to occupy an entire issue—for numerous facts and illustrations could be offered as proofs in support of our position—but we believe we have said enough to show the tenability, the validity of our reasons, the impregnability of our position, to any fairminded person, to any person who really wants to know the truth.

And now for a brief resumé of the conclusions based upon the facts and arguments presented in our editorial. The conclusions are as follows:

1. The products introduced into the Pharmacopeia and National Formulary as substitutes for other well-established products are inferior, in practically every instance, to the originals, while some of the formulas yield nasty, irritating, nauseating and therefore therapeutically worthless products.

2. To urge the physician to prescribe these imitations *in lieu* of the original products, is therefore dishonest. The physician *is not in any way* benefited, while the patient is distinctly injured.

3. This so-called National Formulary Propaganda has nothing to do with ethics. Instead of elevating, it tends, as we have shown, to degrade both pharmacy and medicine. It is purely a money-making proposition.

4. The public is not in any way benefited by this propaganda, for the patient has to pay just as much (and often more), for the *inferior* substitute as for the *superior* original.

5. The deduction which logically and inevitably follows from the above conclusions is this: If you know the composition of a product and that product has given you satisfactory results in your practice, stick to that product; prescribe it and see that you get it; and do not allow yourself by specious reasoning and false claims to be persuaded to use an imitation or a substitute, *be that imitation or substitute official or non-official.*

*Dixi.*

